# Preservation of Distortion Product Otoacoustic Emissions in *OTOF*-Related Hearing Impairment

**DOI:** 10.1097/AUD.0000000000001421

**Published:** 2023-09-06

**Authors:** Rosamaria Santarelli, Pietro Scimemi, Elona Cama, María Domínguez-Ruiz, Chiara Bonora, Chiara Gallo, Montserrat Rodríguez-Ballesteros, Ignacio del Castillo

**Affiliations:** 1 Department of Neurosciences, University of Padua, Padua, Italy; 2 Audiology Service, Santi Giovanni e Paolo Hospital, Venezia, Italy; 3 Servicio de Genética, Hospital Universitario Ramón y Cajal, IRYCIS, Madrid, Spain; 4 Centro de Investigación Biomédica en Red de Enfermedades Raras (CIBERER), Madrid, Spain.

**Keywords:** Auditory neuropathy, Congenital hearing impairment, Distortion product otoacoustic emissions, *OTOF* gene

## Abstract

**Objectives:**

Attenuation of otoacoustic emissions over time has been reported for many patients with hearing impairment harboring mutations in the *OTOF* gene. In this study, the time course of changes of distortion product otoacoustic emissions (DPOAEs) has been analyzed in a cohort of patients in the light of tympanometry results.

**Design:**

The changes of DPOAEs in 16 patients with *OTOF*-related hearing impairment were retrospectively analyzed.

**Results:**

All but one subject showed DPOAEs bilaterally at the time of diagnosis. Three patients diagnosed as adults still had DPOAEs at ages of 27, 31, and 47 years, respectively. Follow-up was available for 7 children diagnosed at the age of 1 to 3 years, who still showed preservation of DPOAEs at ages of 5 to 16 years. The responses were absent or attenuated in amplitude at some follow-up appointments in association with type B or C tympanograms.

**Conclusions:**

DPOAEs are preserved much longer than expected in a cohort of patients with *OTOF*-related hearing impairment. The previously reported loss of DPOAEs may have been caused in some children by increased middle ear impedance due to otitis media.

## INTRODUCTION

Auditory neuropathy (AN) is a hearing disorder characterized by alteration of temporal coding of acoustic signals in the auditory fibers, which results in severe impairment of speech perception and the absence of auditory brainstem responses (Starr et al. 2008; Moser & Starr 2016). In contrast, cochlear receptor outer hair cell (OHC) activities are preserved (otoacoustic emissions [OAEs], cochlear microphonic). The mechanisms believed to be involved are functional alterations at pre- and postsynaptic sites, including neurotransmitter release from ribbon synapses, spike initiation in auditory nerve terminals, loss of nerve fibers and demyelination. The most well-known form of pre-synaptc AN is associated with mutations in the *OTOF* gene (DFNB9) encoding otoferlin, a transmembrane protein involved in glutamate neurotransmitter release (Roux et al. 2006), vesicle replenishment at the inner hair cells ribbon synapses (Pangršič et al. 2010), proper synapse development in immature cochlea and maintenance of outer and inner hair cell function (Stalmann et al. 2021).

Mutations in the *OTOF* gene account for 1.4% to 5% of cases of autosomal recessive nonsyndromic congenital hearing impairment in different populations (Varga et al. 2006; Rodríguez-Ballesteros et al. 2008; Choi et al. 2009; Romanos et al. 2009; Duman et al. 2011; Iwasa et al. 2013). The majority of patients show severe to profound congenital hearing loss, but a temperature-sensitive phenotype and progressive hearing impairment have also been found in association with specific missense mutations (see Varga et al. 2006; Chiu et al. 2010; Wang et al. 2010; Yildirim-Baylan et al. 2014; Vona et al. 2020 for a review). Moreover, stable mild-to-moderate hearing loss associated with severe impairment of speech perception has been reported in some patients (Santarelli et al. 2021).

Over 50% of children with two mutant alleles of *OTOF* gene have a preserved OHC function as indicated by OAEs and cochlear microphonic recordings (Rodríguez-Ballesteros et al. 2008). However, patients with *OTOF* mutations tend to lose OAEs over time (Rodríguez-Ballesteros et al. 2008; Chiu et al. 2010; Yildirim-Baylan et al. 2014; Kitao et al. 2019). Kitao et al. (2019) have reported the results of a 7-year follow-up of 20 hearing-impaired children with biallelic mutations in the *OTOF* gene. They found that 60% of patients showed progressive deterioration of OAEs, with 20% of children losing OAE responses by the age of 2 years. However, there are no studies addressing the causes of OAE loss in *OTOF*-related hearing impairment. In addition, no extended follow-up was performed to check for possible OAE recovery. Specifically, the question of the coexistence of transient OAE loss with conductive hearing impairment due to otitis media with effusion (OME) has not been addressed. OME, which occurs with high frequency in children aged 1 to 4 years, impacts on the amplitude of OAEs recorded in the ear canal, eventually leading to OAEs disappearance (Marcrum et al. 2017). Consequently, the presence of OAEs responses could have been overlooked in some children in the presence of middle ear dysfunction due to tympanic membrane retraction or middle ear effusion.

In this study, we retrospectively analyzed the changes of distortion product otoacoustic emissions (DPOAEs) in a group of 16 patients with hearing impairment due to mutations in the *OTOF* gene in the light of the tympanometry measures collected on the same OAE recording session.

## MATERIALS AND METHODS

### Patients

In this retrospective study, the DPOAE data from *OTOF* patients were extracted from their medical records maintained at the Audiology Service of “Santi Giovanni e Paolo” Hospital in Venice (in accordance with the 1964 Declaration of Helsinki).

DPOAEs had been collected from 16 subjects (age range at the first evaluation 2 months to 47 years, 8 females) with hearing impairment due to biallelic mutations in the *OTOF* gene at each follow-up appointment throughout the last 20 years. Genetic and audiological data have been previously reported (Santarelli et al. 2015, 2021) except for subjects nos. 7, 8 and 16, who were diagnosed later.

The details of genetic findings together with audiological and electrophysiological measures for all subjects are summarized in Table 1. Subjects nos. 4, 5, and 6 were brothers and sister. Subjects nos. 1, 2 and 16 were diagnosed in their adult life. Specifically, subjects nos. 1 and 2 had undergone several audiological assessments in their childhood, but the identification of the auditory neuropathy profile and the genetic diagnosis were established at the age of 22 and 27 years, respectively. Subject no. 16 received clinical and genetic diagnosis at the age of 47 years.

**TABLE 1.** Genetic and audiological findings from *OTOF* patients

Subjects	Gender	Genotype	ACMG Class	Age at Diagnosis	Hearing Loss	PTA (dB) R/L	Acoustic Reflexes	ABRs	CI	Age at CI
No. 1	F	c.3127-1G>A/c.1469C>G (p.Pro490Arg)	P/LP	22 yrs	Mild/mild	25/20	**+/+**	abs/V	-	
No. 2	F	c.5819C>G (p.Pro1940Arg)/c.5819C>G (p.Pro1940Arg)	LP/LP	27 yrs	Mild/mild	40/36	**+/+**	abs/abs	CI24RECA	28 yrs
No. 3	M	c.1601delC (p.Pro534Glnfs*4)/c.2732_2735dup (p.Tyr913Alafs*90)	P/P	20 mo	Profound	114	abs/abs	abs/abs	CI24RECA	24 mo
No. 4	M	c.2732_2735dup (p.Tyr913Alafs*90)/c.2891C>A (p.Ala964Glu)	P/P	27 mo	Profound	102	abs/abs	abs/abs	CI24RECA	27 mo
No. 5	F	c.2732_2735dup (p.Tyr913Alafs*90)/c.2891C>A (p.Ala964Glu)	P/P	8 mo	Profound	109	abs/abs	abs/abs	CI24RECA	19 mo
No. 6	M	c.2732_2735dup (p.Tyr913Alafs*90)/c.2891C>A (p.Ala964Glu)	P/P	8 mo	Profound	110	abs/abs	abs/abs	CI512	13 mo
No. 7	M	c.4275G>A (p.Trp1425*)/c.4275G>A (p.Trp1425*)	P/P	21 mo	Profound	111	abs/abs	abs/abs	CI512	26 mo
No. 8	F	c.2887C>T (p.Arg963*)/c.620_623dup (p.Ala209Serfs*25)	P/P	29 mo	Profound	105	abs/abs	abs/abs	CI512	33 mo
No. 9	F	c.5452G>T (p.Asp1818Tyr)/c.5792C>T (p.Pro1931Leu)	LP/LP	5 yrs	Mild/mild	26/21	abs/abs	abs/V	CI532	6 yrs
No. 10	F	c.1601delC (p.Pro534Glnfs*4)/c.1694T>C (p.Phe565Ser)	P/LP	36 mo	Moderate	51	abs/abs	abs/abs	CI512	41 mo
No. 11	M	c.5900_5902del (p.Ile1967del)/c.5401dup (p.Ala1801Glyfs*41)	LP/P	18 mo	Mild	27	**+/+**	abs/abs	-	
No. 12	M	c.5384T>G (p.Phe1795Cys)/c.5384T>G (p.Phe1795Cys)	LP/LP	31 mo	Profound	114	abs/abs	abs/abs	Yes	
No. 13	M	c.3400C>T (p.Arg1134*)/c.5217G>A (p.Trp1739*)	P/P	18 mo	Profound	107	abs/abs	abs/abs	Yes	
No. 14	F	c.1609delG (p.Val537*)/c.1966delC (p.Arg656Glyfs*10)	P/P	15 mo	Profound	120	abs/abs	abs/abs	CI24RECA	46 mo
No. 15	F	c.2239G>T (p.Glu747*)/c.2239G>T (p.Glu747*)	P/P	26 mo	Profound	107	abs/abs	abs/abs	CI24RECA	28 mo
No. 16	M	c.2071_2081del (p.Met691Glyfs*63)/c.3733 + 1G>A	P/P	47 yrs	Moderate	44/47	**+/+**	abs/abs	-	

published online ahead of print September 6, 2023.

+, present; abs, absent; ABRs, auditory brainstem responses; ACMG, American College of Medical Genetics and Genomics; CI, cochlear implantation; LP, likely pathogenic; P, pathogenic; PTA, pure-tone average (average thresholds at 0.5, 1, 2, 4 kHz); R/L, right ear/left ear.

The degree of hearing impairment was defined by the pure-tone average (PTA) threshold levels measured at 0.5, 1, 2, and 4 kHz, and was classified as mild (PTA 21 to 40 dB HL), moderate (PTA 41 to 70 dB HL), severe (PTA 71 to 95 dB HL), and profound (PTA > 95 dB HL) (Martini et al. 1997). Profound deafness was found in 10 patients, 4 had mild hearing impairment while 2 patients showed moderate hearing threshold elevation. Hearing thresholds were assessed at each follow-up appointment and remained stable over time in all patients.

Computed tomography (CT) and magnetic resonance imaging (MRI) scans of the head and ear as well as growth and motor development were normal in all subjects. No patient had a history of sound exposure.

All but 3 patients received unilateral cochlear implant in the right year, except for subject no. 15, who was implanted in the left ear because of persistent OME in the right ear. Nine patients attempted a trial with hearing aids, but only 4 subjects (nos. 3, 4, 12, 14) underwent a course longer than 1 year. One patient (no. 3) was still wearing the hearing aid in the nonimplanted ear at the last follow-up appointment.

It is noteworthy that 11 (nos. 3, 4, 5, 6, 7, 8, 9, 10, 13, 14, 15) of 16 patients showed OME at least at one follow-up appointment, which was diagnosed based on otoscopic evaluation and tympanometry recording (Grason-Stadler GSI TympStar or Madsen Zodiac impedance audiometer). Tympanogram was indicated as type A, As, B, or C according to Jerger (1970) classification. The normal range of peak compliance was 0.2 to 0.9 mm H_2_O in children and 0.3 to 1.4 mm H_2_O in adults (Margolis & Heller 1987).

### DPOAEs Recordings

DPOAEs were obtained using an ILO-92 system up to 2018 (subjects nos. 3, 11, 12, 13, 14, 15) and an ILO-292 OAE (all the remaining subjects, except for the first recording session in subjects nos. 5, 6, 7, 10) system thereafter. Primary tones were presented at 65/55 dB SPL and the *f*2/*f*1 ratio was kept at 1.21. The *f*2 frequency was increased in 1/4 octave steps from 1.0 to 8.0 kHz (ILO-92 system) or to10.0 (ILO-292 system).

At each *f*2 frequency, DPOAEs were considered present when the signal to noise ratio was higher than 3 to 6 dB (Audiologic Guidelines for the Assessment of Hearing in Infants and Young Children, American Academy of Audiology 2020). DPOAEs rate was defined on the basis of the percentage of frequency points showing a positive response as reported by Kitao et al. (2019).

## RESULTS

**Fig. 1.**
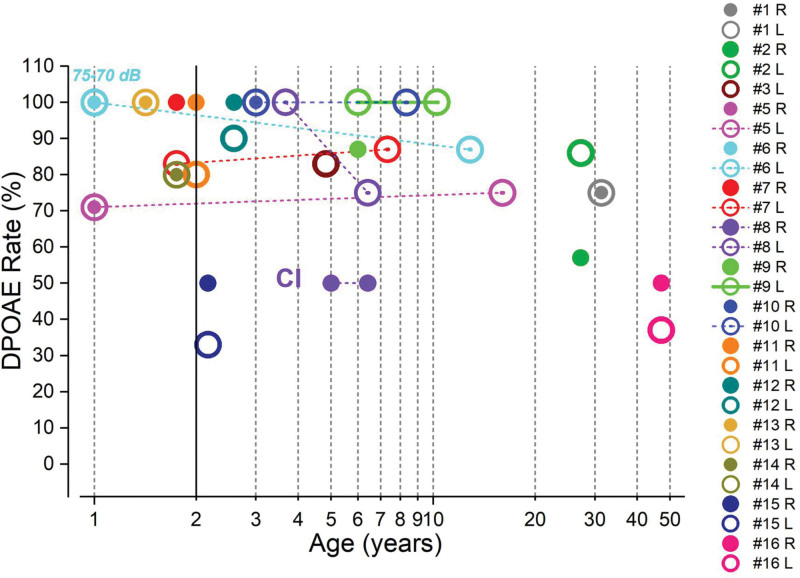
DPOAEs rates at the first and last follow-up appointments in the presence of normal tympanometry in patients with *OTOF*-related hearing impairment. Response rate was calculated as the percentage of frequency points showing a positive response. The DPOAEs recordings collected at ages of 2 and 5 mo in subjects nos. 5 and 6 were reported at the age of 1 for the sake of clarity. In this and in the subsequent figures, DPOAEs were recorded at 65/55 dB SPL intensity, except for the responses collected in subject no. 6 at the age of 5 mo, which were obtained at 75/70 dB SPL. DPOAEs indicate distortion product otoacoustic emissions; L, left ear; R, right ear.

Of the 16 patients included in this study, only one (subject no. 4) lost OAEs few months after birth. DPOAEs rates at the first and last follow-up appointments in the presence of normal tympanometry (type A or type As, compliance ≥2 mmho) are reported in Figure 1 for the other 15 patients. In 5 subjects (subjects nos. 11 to 15), DPOAEs were recorded up to the age of 2 to 3 years. Of these, 4 were affected by profound hearing loss and one had mild hearing impairment (Table 1). No further DPOAE recordings are available, since parents chose to refer to other institutions. These children had robust DPOAEs responses, except for subject no.15 whose OAE recordings, however, showed high noise levels.

Four patients (subjects nos. 3, 5, 6, 7) with profound hearing loss have been following up since the age of 1 to 2 years. They had DPOAEs in both ears before cochlear implantation and still showed DPOAEs in the nonimplanted ear at the age of 4.8, 16, 12.8, and 7.3 years, respectively. It is interesting that subject no. 3 still had DPOAEs at the age of 4.8 in the nonimplanted ear where he was wearing a power hearing aid since the age of one.

The children diagnosed after the age of 3 (subjects nos. 8, 9, 10) still had DPOAEs in the nonimplanted ear at age of 6.4, 10.2, and 8.3 years, respectively. It is of note that in subject no. 8, the DPOAEs responses were recorded at the age of 6.4 also in the implanted ear. Of these patients, subject no. 8 showed profound deafness, whereas subjects nos. 9 and 10 had, respectively, mild and moderate hearing loss.

In all subjects who underwent an extended follow-up the hearing thresholds measured in the nonimplanted ear remained stable over time.

The 3 patients (subjects nos. 1, 2, 16) diagnosed as adults still had DPOAEs at ages of 27, 31, and 47 years, respectively. The degree of hearing impairment was mild in subjects nos. 1 and 2, and moderate in subject no. 16 (Table 1).

**Fig. 2.**
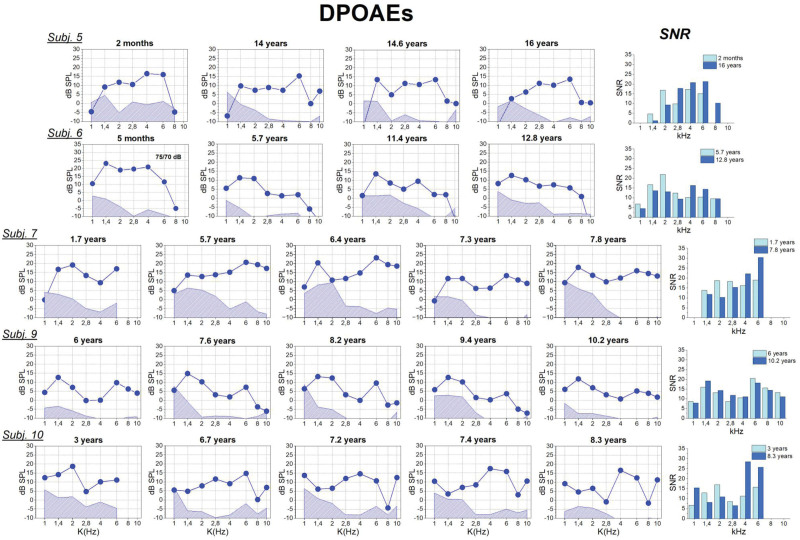
DPOAEs recordings collected in 5 subjects (left ear) in the presence of normal tympanometry at different ages. For each patient, the SNR at each frequency was compared between the first and last appointment in the graph reported on the left. Subjects nos. 5, 6, and 7 were affected by profound deafness, whereas subjects nos. 9 and 10 had, respectively, mild and moderate hearing loss. DPOAEs indicate distortion product otoacoustic emissions; SNR, signal to noise ratio.

Figure 2 shows the DPOAEs responses collected in 5 subjects (non-implanted ear) in the presence of normal tympanometry (type A, compliance ≥2 mmho) at different times after the first recording session. For all patients, the signal to noise ratio measured at each frequency was compared between the first and last appointment. DP-grams show that the amplitude of OAEs responses was substantially stable over time.

**Fig. 3.**
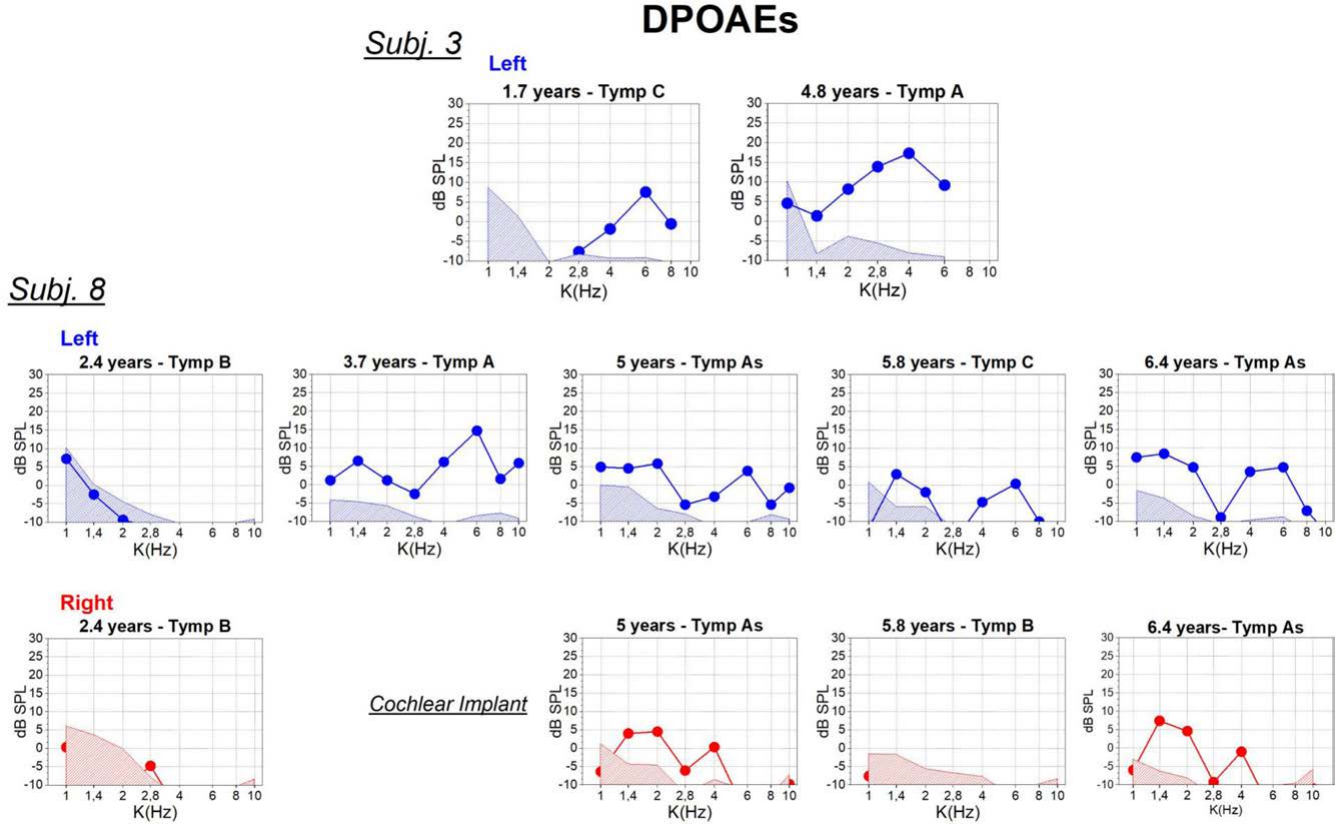
DPOAEs responses recorded at different ages in two patients with *OTOF*-related hearing impairment who suffered from recurrent episodes of OME. The type of tympanogram (A, As, B, C) was indicated for each DPOAE recording according to Jerger (1970) classification. Type B and C tympanograms were associated, respectively, with absence or attenuation of DPOAE responses. DPOAEs indicate distortion product otoacoustic emissions; OME, otitis media with effusion.

DPOAEs recordings collected in 2 subjects (nos. 3, 8) at follow-up appointments during which tympanometry indicated the presence of increased middle ear impedance are displayed in Figure 3. The DPOAEs recorded from subject no. 3 (non implanted ear) at the age of 1.7 years in the presence of type C tympanogram were of low amplitude and were confined to the high-*f*2 frequency range. In contrast, DPOAEs appeared remarkably increased in amplitudes and became apparent also at low *f*2 frequencies at the age of 4.8 years, when the recurrent episodes of OME had faded away. In subject no. 8, the presence of DPOAEs at different follow-up appointments was strictly dependent on the compliance of the middle ear. Indeed, DPOAEs were recorded only in the presence of type A or As tympanogram, while type B and C tympanograms were associated, respectively, with the absence or attenuation of DPOAE responses. It is of note that DPOAEs responses were recorded also in the implanted ear in the presence of type As tympanogram.

**Fig. 4.**
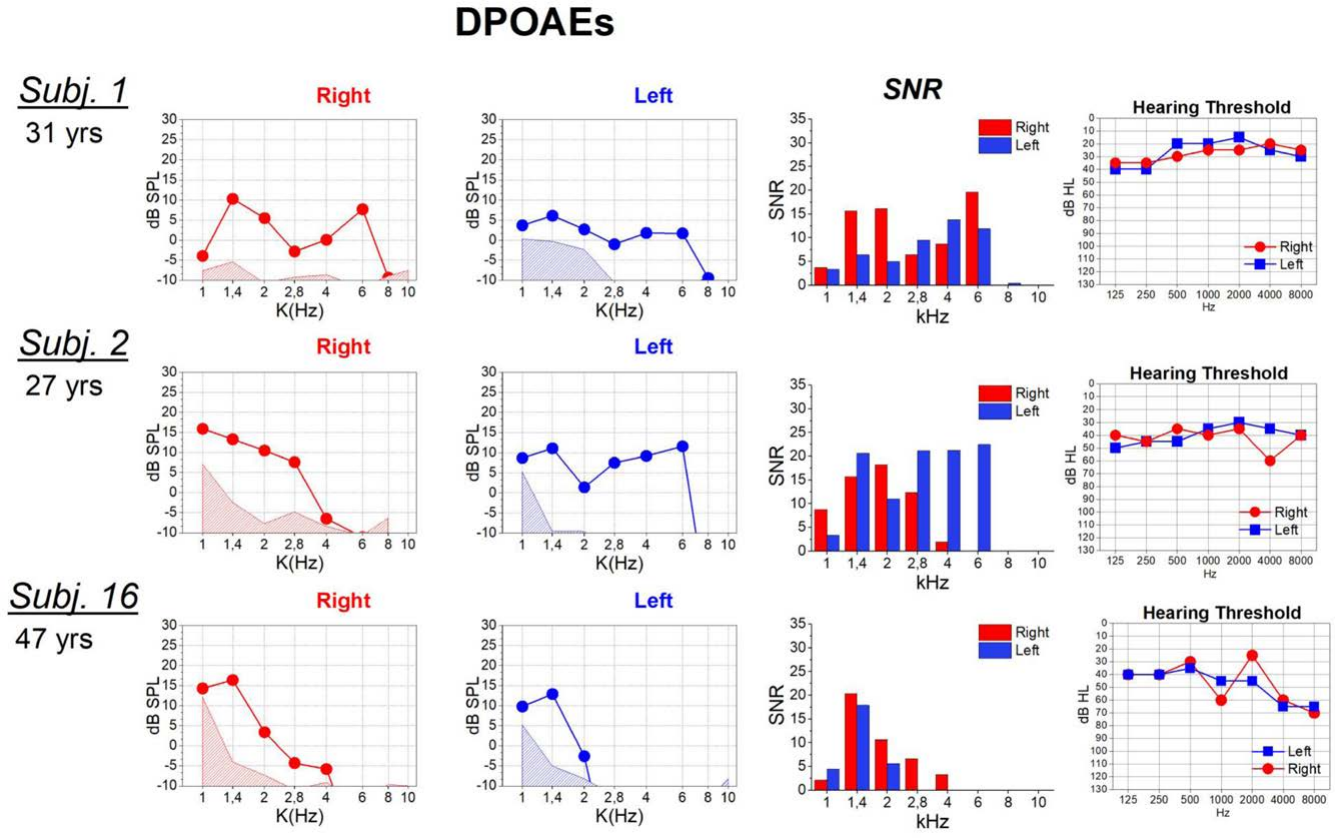
DPOAEs responses recorded in 3 adult patients together with the SNR at each frequency. On the left, the hearing thresholds obtained on the same session of DPOAEs recording are shown. SNR, signal to noise ratio.

DPOAEs collected in the 3 patients diagnosed as adults are shown in Figure 4 together with the hearing thresholds measured on the same session. Type A tympanogram was obtained on each session. All subjects showed DPOAEs responses in both ears. However, subject no. 2 failed to show DPOAEs at high *f*2 frequencies, while subject no. 16 showed robust DPOAEs only at *f*2 frequencies lower than 2.8 kHz. This patient also had elevated hearing thresholds at high frequencies.

In conclusion, the 10 patients diagnosed and/or followed up after the age of 3 years showed DPOAEs in both ears (nos. 1, 2, 8, 16) or in the nonimplanted ear (nos. 3, 5, 6, 7, 9, 10) at the last follow-up appointment (age range 4.8 to 47 years).

## DISCUSSION

The findings reported in this study indicate preservation of OAEs in a cohort of patients with *OTOF*-related hearing impairment. The disappearance of OAEs responses or their attenuation in amplitude in some children was associated with the coexistence of OME as indicated by recording type B or C tympanogram.

Previous studies have reported the absence or deterioration of OAE responses in patients harboring pathogenic mutations in the *OTOF* gene (Rodríguez-Ballesteros et al. 2008; Chiu et al. 2010; Yildirim-Baylan et al. 2014; Kitao et al. 2019). However, most studies report single observations and are rarely associated with tympanometry recordings. A follow-up evaluation of DPOAEs responses has been reported only by Kitao et al (2019), who found that OAEs decreased in amplitude in 12 of 20 ears (60%) of their sample, with the majority of children losing OAE responses by the age of 2 years. Nevertheless, the authors reported that tympanometry was performed in these patients only when OME was suspected based on otoscopy, and that changes in middle ear transmission could have been overlooked in some children. In our study, none of the 7 patients followed up for many years showed deterioration of DPOAEs, since all continued to show robust DPOAE responses up to the age of 16. In addition, the 3 patients diagnosed as adults (subjects nos. 1, 2, 16) still had DPOAEs at the last follow-up appointment.

Of the patients included in this study, subjects with profound deafness had inactivating mutations in both *OTOF* alleles, whereas mild phenotypes correlate with the presence of at least one missense variant or one in-frame deletion. Although a limited number of patients were considered, the genotype does not seem to affect OAEs changes in most *OTOF* subjects, since OAEs were preserved in the 5 children with profound deafness, harboring inactivating mutations, as well as in subjects who carried at least one missense mutation resulting in a partially functional protein and mild-to-moderate hearing loss. Moreover, of the 3 siblings with profound deafness having the same genotype (subjects nos. 4, 5 and 6), one lost DPOAEs by the age of one year, whereas the remaining two showed preservation of OAE responses up to the age of 12.8 and 16 years, respectively.

Previous studies have reported that the amplitude of DPOAEs show significant differences between infants and adults (Prieve et al. 1997; Lasky 1998), the infants showing higher mean DPOAE levels for *f*2 frequencies ranging from 1500 to 5000 Hz in comparison with teens and adults. These differences are deemed to result from the developmental changes occurring in the outer and middle ear (Lasky 1998; Abdala & Keefe 2006). However, it is difficult to assess whether and to what extent these developmental changes had impinged on the DPOAEs amplitude measured in patients who underwent an extended follow-up, due to the small number of subjects followed up since the age of 1 to 3 years. Nevertheless, DPOAEs recordings collected in individual patients at different ages point to substantial preservation of the OHC function.

The 3 adults showed DPOAEs in both ears. Nevertheless, 2 patients (subjects nos. 2, 3) had no DPOAEs at high *f*2 frequencies, while subject no. 16 showed robust responses only at low *f*2 frequencies. This finding could indicate an age-dependent deterioration of OHCs, starting from the base and progressing toward the apex of the cochlea, which accords with the findings reported by Stalmann et al. (2021) in *OTOF* knock-out mice. Overall, the findings reported in this study may indicate that OAEs deterioration besides being age-dependent, begins at an older age than previously reported, at least in some patients.

In some children, DPOAEs disappeared or were markedly attenuated in amplitude in the presence of an increased middle ear impedance due to OME even in cases of a minimal decrease of the peak compliance in tympanometry. Of note, in one child (subject no. 3), DPOAE amplitudes appeared markedly increased at the age of 5 in comparison to previously recorded DPOAEs, probably because the episodes of OME had faded away. Based on these findings, it seems conceivable that in some of the previously published studies, the presence of DPOAEs in *OTOF* patients suffering from recurrent episodes of OME could have been missed, particularly in those children for whom an extended follow-up was not available. We believe that, at least in some patients, the OAEs presence has been underestimated as the attenuation in amplitude could have resulted from an increased middle ear impedance due to OME rather than from OHC loss.

Another point to be addressed when investigating the changes of OAEs in *OTOF*-related hearing impairment deals with the high noise levels often observed in highly active toddlers, which could mask positive OAE responses. An example is given by the DPOAEs obtained in subject no. 15, for whom only noisy recordings were available. These cases require an extended follow-up to select the data collected in the best recording condition.

As bilateral cochlear implantation is the standard of care for children with severe to profound hearing loss, it is likely that profoundly-deaf children harboring mutations in the *OTOF* gene received the bilateral cochlear implant by 1 to 2 years of age. This could have been a critical factor in preventing an extended follow-up of DPOAEs recordings in these patients. All but 2 subjects of our sample received unilateral cochlear implant, which allowed us to extend the DPOAE follow-up after cochlear implantation. It is interesting that low-amplitude DPOAEs were recorded also in the implanted ear in one subject (no. 8) at the appointments in which type A tympanogram was obtained. This finding points to preservation of OHCs, which survived to the trauma induced by the insertion of the cochlear implant array.

When considering the hearing aid use, only 4 subjects used hearing aids for more than one year, whereas the remaining patients tended to use acoustic amplification discontinuously. It is interesting that subject no. 3, who was wearing one power hearing aid in the nonimplanted ear since the age of one, still had robust DPOAEs at the age of 5 years. Nevertheless, due to unavailability of an extended follow-up for the other patients, the effects of amplification on DPOAEs cannot be assessed.

In conclusion, this study demonstrates that DPOAEs are preserved much longer than expected in a cohort of patients with *OTOF*-related hearing impairment. Although other unidentified genetic or environmental modifiers may be responsible for the early OAE loss in a minority of patients, the disappearance or the decrease of OAE amplitude in some patients could have been induced by an increase of middle ear impedance due to OME or to high noise levels during OAEs recording. These findings are relevant not only from the point of view of the elucidation of the mechanisms underlying hair cell damage induced by otoferlin deficiency, but mainly because they indicate preservation of the cochlear amplifier, which is crucial for the full restoration of hearing through gene therapy administration.

## ACKNOWLEDGMENTS

We are greatly indebted to Drs. Alessandra Ferraboschi, Serena Scardillo, Marica Pistello who helped with data collection.
